# Grain-Based Dietary Background Impairs Restoration of Blood Flow and Skeletal Muscle During Hindlimb Ischemia in Comparison With Low-Fat and High-Fat Diets

**DOI:** 10.3389/fnut.2021.809732

**Published:** 2022-01-10

**Authors:** Iurii Stafeev, Maria Boldyreva, Svetlana Michurina, Elizaveta Mamontova, Elizaveta Ratner, Mikhail Menshikov, Yelena Parfyonova

**Affiliations:** ^1^National Medical Research Center for Cardiology, Moscow, Russia; ^2^Faculty of Biology and Biotechnology, National Research University Higher School of Economics, Moscow, Russia; ^3^Lomonosov Moscow State University, Moscow, Russia

**Keywords:** hindlimb ischemia, insulin resistance, muscle regeneration, grain-based diet, high-fat diet, obesity

## Abstract

**Background:** Among vascular pathologies associated with obesity, peripheral artery disease (PAD) occupies the important position. In clinical practice, nutritional interventions are recommended for patients with PAD. In this work, we investigated how the different dietary backgrounds affect the regeneration rate of ischemic hindlimb in mice.

**Methods:** Male C57BL/6J mice were housed on three types of diet: low-fat (LFD), high-fat (HFD), and grain-based diet (GBD) for 13 weeks. Metabolic parameters including FBG level, ITT, and GTT were evaluated. The blood flow was assessed by laser Doppler scanning on 7, 14, and 21 days after hindlimb ischemia. Necrotic area of m.tibialis, macrophage infiltration, and angiogenesis/arteriogenesis were evaluated by histology. Glucose uptake in recovered skeletal muscle was analyzed using [3H]-2-deoxyglucose, and GLUT1 and GLUT4 expression were assessed by Western blotting.

**Results:** In our work, we developed three experimental groups with different metabolic parameters: LFD with normal glucose metabolism, GBD with mild hyperglycemia, and HFD with impaired glucose tolerance. GBD-fed mice had a tendency to increase necrosis of *m. tibialis* and significantly higher macrophage infiltration than LFD and HFD groups. Moreover, GBD-fed mice had a trend to decreased blood flow recovery and significantly impaired arteriogenesis. Recovered skeletal muscle of GBD-fed mice had lower glucose uptake and decreased level of GLUT4 expression.

**Conclusion:** Thus, we conclude that dietary background and metabolic status determine the rate of post-ischemic regeneration including angiogenesis, skeletal muscle recovery and metabolic activity. The most effective regeneration is supported by LFD, while the lowest rate of regeneration occurs on GBD.

## Highlights

- HFD has impaired glucose tolerance and GBD has impaired fasting blood glucose
- GBD has highest necrotic area and macrophage infiltration of skeletal muscle
- Blood flow recovery is impaired in GBD-fed mice through attenuation of arteriogenesis
- Skeletal muscle glucose uptake and GLUT4 expression are impaired on GBD
- GBD has decreased regeneration rate in hindlimb ischemia model

## Introduction

Diet and nutrition have a crucial impact on whole body metabolism and physiology. Caloric overload and consumption of food with high fat, sucrose, or fructose content leads to weight gain, obesity, and metabolic abnormalities (1–4). According to recent reports, the prevalence of obesity and associated pathologies such as insulin resistance, type 2 diabetes, atherosclerosis, and peripheral artery disease (PAD) significantly increased during the last decades (5–9). Furthermore, the course of micro- and macrovascular abnormalities is complicated in obese and diabetic states by impaired wound healing (10, 11). Here, we investigate the effect of a high fat diet compared with a low fat/refined grain diet and a medium fat/whole grain diet on insulin sensitivity and hindlimb recovery.

The regeneration of muscles after ischemia depends on several aspects: restoration of blood flow, removal of damaged cells, and resolution of inflammation, allowing myogenic stem cells proliferation and differentiation. Blood flow restoration also depends on microenvironmental cues (inflammation and oxidative stress), including angio- and arteriogenesis, and reperfusion of novel vessels (12).

High-fat diet (HFD) has a strong influence on post-ischemic regeneration. Previous studies reported suppressed recovery of muscles in animals on HFD in comparison with LFD (13–15). Increase of circulating fatty acids activates phosphatase PTEN and inhibits AMP-dependent kinase, dampening myofibers maturation (13, 14). Regenerated muscles of mice on HFD have lower functional characteristics even long after injury (15, 16). Furthermore, HFD has an effect on microvascular remodeling and endothelial function. HFD-induced insulin resistance impairs microvascular function by lowering endothelial cell nitric oxide production, resulting in reduced vasodilation and secretion of proangiogenic factors (17, 18). Obese individuals also exhibit chronic low-grade inflammation and oxidative stress, conditions inhibiting restorative phase of regeneration (16, 19–23). However, the recent study reports controversial results, suggesting the positive role of HFD in post-ischemic regeneration (24).

Low-fat and whole grain-based diets (LFD and GBD, respectively), are predominant dietary interventions aimed at prevention of obesity complications and cardiovascular diseases (25–29). Indeed, LFD improves the prognosis of PAD due to the restoration of blood flow and vascular health (30, 31). In contrast, the role of GBD in a post-ischemic regeneration remains not well-characterized. GBD demonstrates therapeutic potential in liver regeneration and therapy of fatty liver diseases (32, 33). Moreover, wheat and other whole grains promote survival and proliferation of progenitor cells (34, 35). On the other hand, whole grains cause reduction of angiogenesis and are used as a part of antiangiogenic strategy in cancer therapy (36, 37).

The lack of studies on the role of GBD in post-ischemic regeneration encouraged us to investigate its potential for the improvement of hindlimb post-ischemic recovery. The comparison of muscle and vascular regeneration on LFD, GBD, and HFD will help to distinguish the beneficial diet and to suggest interconnections between metabolism and regeneration.

## Methods

### Animals and Diets

Five-week old male C57BL/6J mice (purchased from “Andreevka” animal husbandry facility, Russia) were matched by body weight and fasting blood glucose (FBG) level (body weight reference range was from 17 to 23 g; FBG reference range was from 3.9 to 6.9 mM). At the age of 6 weeks mice were randomized into 3 groups (7–8 animals per group) and a diet experiment was started. Mice were kept under standard pathogen-free conditions with food and water *ad libitum* and regular 12:12 light–dark cycle. All diets were purchased from manufacturers: low-fat diet (LFD) D12450H, “Research Diets,” USA; grain-based diet (GBD) PK120, “Gatchina Animal Complete Feeds Factory,” Russia; high-fat diet (HFD) D12451, “Research Diets,” USA. More information about protein/fat/carbohydrates content and raw components of diets are presented in Supplementary Tables 1, 2. The mice were put on LFD, GBD, and HFD for 10 weeks. During the experiment body weight, FBG and food consumption were measured every 2 weeks. For the FBG measurements glucometer Contour Plus One, “Ascencia Diabetes Care,” Switzerland was used. Brief description of the study design is presented in Supplementary Figure 1. This work can be accounted as an explorative study and contains multiple outcomes. No power calculation was used to determine the sample size because at the start of our work as we did not have the data for expectative range of outcomes, neither in our previous experiments nor in the previous works. The animal experiment was performed in accordance with the EU Directive 2010/63/EU for animal experiments and approved by Institutional Ethics Board for Animal Care (National Medical Research Center for Cardiology, permit #385.06.2009).

### Intraperitoneal Glucose Tolerance and Insulin Tolerance Tests

Glucose tolerance test (GTT) and insulin tolerance test (ITT) were performed at the 10th week of experimental feeding. Mice were fasted overnight and weighted before measurements. Blood samples were collected from the tail vein by cutting the tip of the tail by sterile scalpel; blood glucose level was measured with glucometer. For GTT, 2 g/kg of glucose was injected intraperitoneally in the form of a 10% sterile glucose solution. The blood glucose level was measured before glucose injection (0 min) and in 15, 30, 45, 60, and 90 min after glucose administration. However, GTT does not allow to distinguish insulin resistance in peripheral tissues and reduced capacity of the pancreatic β-cells to insulin production. To resolve this problem, we also performed ITT, where the plasma glucose disappearance rate indicates whole-body insulin sensitivity and correlates well with the gold-standard glucose clamp technique (38). For ITT, 0.5 IU/kg of human insulin (Humulin, “Lilly France GmbH,” France) was injected intraperitoneally. The blood glucose level was measured before insulin injection (0 min) and in 15, 30, 45, 60, 90, 120, 150, and 180 min after insulin administration.

### Hind Limb Ischemia Modeling and Postsurgical Care

After 10 weeks of experimental feeding all mice underwent surgical modeling of hind limb ischemia, which can serve as a PAD animal model (39, 40). Mice were anesthetized by intraperitoneal injection of 2.5% avertin solution. All surgical manipulations were carried out in aseptic conditions under a binocular microscope Leica M620 TTS (“Leica Microsystems,” Germany). Unilateral induction of hind limb ischemia was performed as previously described (41). Briefly, skin was incised along the midline of the left hind limb and the femoral artery, with its branches, and was ligated between its proximal part and popliteal bifurcation. The blood vessel was excised between the upper and lower ligatures with the sciatic nerve kept intact. After that the skin was closed with 5–0 silk sutures, and animals were placed in a chamber on a heated pad until full recovery. After surgery, all animals received a 1.5 mL bolus of warm sterile saline subcutaneously to compensate for blood loss. Postsurgical care included the saving of pre-surgical diet type, standard pathogen-free conditions, and regular 12:12 light-dark cycle. Moreover, laser Doppler perfusion measurement was performed immediately after surgery (0 day) and on days 7, 14, and 21 post-surgery. At week 13, we carried out postsurgical GTT and ITT as described above. After that mice were sacrificed by lethal isoflurane inhalation, skin was dissected, and samples of inguinal fat and ischemic skeletal muscle *m. Tibialis anterior* were collected and frozen in liquid nitrogen.

### Laser Doppler Perfusion Measurement

For the subcutaneous blood flow recovery assessment in ischemic hind limb, we used laser Doppler scanner (“Moor Instruments Ltd,” UK). Animals were anesthetized by avertin intraperitoneal injection as described above. Perfusion was measured (*n* = 3–4) on the plantar surface of the animal's feet, and the obtained data were analyzed by Moor Image Review software. Scans took up to three consequent measurements with minimal variations (<10%). Obtained results were normalized on blood flow in the intact limb and presented as a relative perfusion (%).

### Histological Methods and Morphometry

After skin dissection, the femoral quadriceps and *m.Tibialis anterior* were harvested and frozen in TissueTek medium (Sakura Finetek, Netherlands). Tissue sections (7 μm thick) were prepared on glass slides and stored at −80°C.

#### Hematoxylin/Eosin Staining

Tissue sections were fixed in 4% paraformaldehyde and washed with distilled water. Staining by Mayer's Hematoxylin was performed during 1–2 min and dye was differentiated in flowing water (1 min). After that the slides were stained in eosin B solution (3 min). The slides were washed with 70% ethanol and incubated subsequently in 96% ethanol, 100% ethanol and xylene (10 min). Slides were mounted in Cytoseal-60 (“Richard-Allen Scientific,” USA) under coverslips. Staining was visualized on scanning microscope Leica ScanScope CS, “Leica Microsystems,” Germany and analyzed by Aperio ImageScope software.

#### Immunofluorescent Staining

Tissue sections were fixed in 4% paraformaldehyde solution, washed, and incubated in blocking solution (2% bovine serum albumin; 10% of secondary antibody's donor serum; phosphate buffer solution). Slides were incubated overnight with primary antibodies (anti-CD68, #137001, “Biolegend,” USA; anti-CD31, 550274, “BD Biosciences Pharmingen,” USA; anti-α-smooth muscle actin (α-SMA), ab5694, “Abcam,” UK). After incubation with primary antibodies, the sections were stained with AlexaFluor488-conjugated secondary antibody (#A21206, “Thermo Scientific,” USA) or with AlexaFluor594-conjugated secondary antibody (#A11032, “Thermo Scientific,” USA); all slides were counterstained with DAPI (“Sigma-Aldrich,” USA). Staining was visualized on a fluorescent microscope Zeiss AXIO Observer A1 (Zeiss, Germany) and analyzed by ImageJ freeware. Morphological evaluations were performed on Day 21 after surgery or on the 13th week of diet.

### *Ex vivo* Glucose Uptake

Skeletal muscle biopsies were harvested from the femoral quadriceps. Samples were immediately transferred to warm DMEM with 1 g/l glucose supplemented with 0.1% BSA and incubated at 37°C, 5% CO_2_ for 3 h. Explants were minced into fine pieces and washed twice in DMEM with no glucose; further manipulations were performed in DMEM with no glucose. Insulin was added for 30 min and glucose transport was initiated by the addition of 100 μM of 2-deoxyglucose (Sigma-Aldrich, USA) and 0.2 uCi/ml of [^3^H]-2-deoxyglucose (#ART0103B, American Radiolabeled Chemicals, USA) for 20 min. Uptake was terminated with three washes with ice-cold DPBS, after which the samples were lysed in RIPA buffer (50 mM Tris-HCl, pH 8.0, 150 mM NaCl, 1% Triton X-100, 0.5% sodium deoxycholate, 0.1% sodium lauryl sulfate). Samples were added into Beckman ReadySolv HP scintillation fluid (Beckman, USA). The radioactivity was measured with a RackBeta counter (LKB Wallac, Sweden), and the results were protein-normalized (DC Protein Assay Kit, Bio-Rad, USA).

### Western Blotting

Skeletal muscle biopsies from the femoral quadriceps were frozen in liquid nitrogen and pulverized in liquid nitrogen using porcelain mortar and pestle in 1 μl of RIPA buffer supplemented with proteases (cOmplete Tablets; Roche, Germany) and phosphatases (10 mM sodium glycerophosphate, 20 mM sodium pyrophosphate, 10 mM sodium fluoride, 1 mM sodium orthovanadate) inhibitors per 1 mg of tissue. The extracts were heated for 30 min at 56°C in a sample buffer (65.8 mM Tris-HCl, pH 6.8, 2.1% SDS, 26.3% (w/v) glycerol, 0.01% bromophenol blue), separated by Laemmli SDS-PAGE, and then the proteins were transferred to polyvinylidene difluoride membrane. Membranes were blocked in 5% solution of fat-free milk on TBS containing 0.1% Tween 20 (TBST) and incubated with the primary and secondary antibodies in 1% milk in TBST in dilutions suggested by the manufacturer. We used primary antibodies: anti-GLUT1 (ab652, Abcam, UK), anti-GLUT4 (ab166704, Abcam, UK), and anti-GAPDH (sc25778, Santa Cruz, USA). We used secondary antibodies: anti-rabbit IgG (ab6721, Abcam, USA) and anti-mouse IgG (#115-035-146, Jackson ImmunoRes, USA) conjugated with horseradish peroxidase. The protein bands were visualized using Clarity ECL kit (BioRad, USA) and Fusion FX gel-documentation system (Vilber-Lourmat, France) in the video mode to ensure that digital results are in the linear range. Quantification was performed using the GelAnalyzer2010 software.

### Statistical Analysis

Data is expressed as mean ± standard error mean (SEM) for each group. Software GraphPad Prism 8.0 was used for the statistical analysis and graphs imaging. At each time point, we compared 3 groups (LFD, HFD, and GBD) and did not analyze the difference between time points. AUCs were calculated by Gagnon's method (42). Significance of multiple differences (3 groups) was calculated by Kruskal–Wallis test with statistical significance threshold set at *p* < 0.05. Additionally, significant differences between the groups were found, a *post-hoc* Dunn's multiple comparison test was performed, and statistical significance threshold also set at *p* < 0.05.

## Results

### Both HFD and GBD Impair Glucose Tolerance in Mice

The insulin sensitivity and glucose tolerance of mice fed LFD, GBD, or HFD were estimated by analyzing body weight, FBG, GTT, and ITT at week 10.

**Figure 1:**
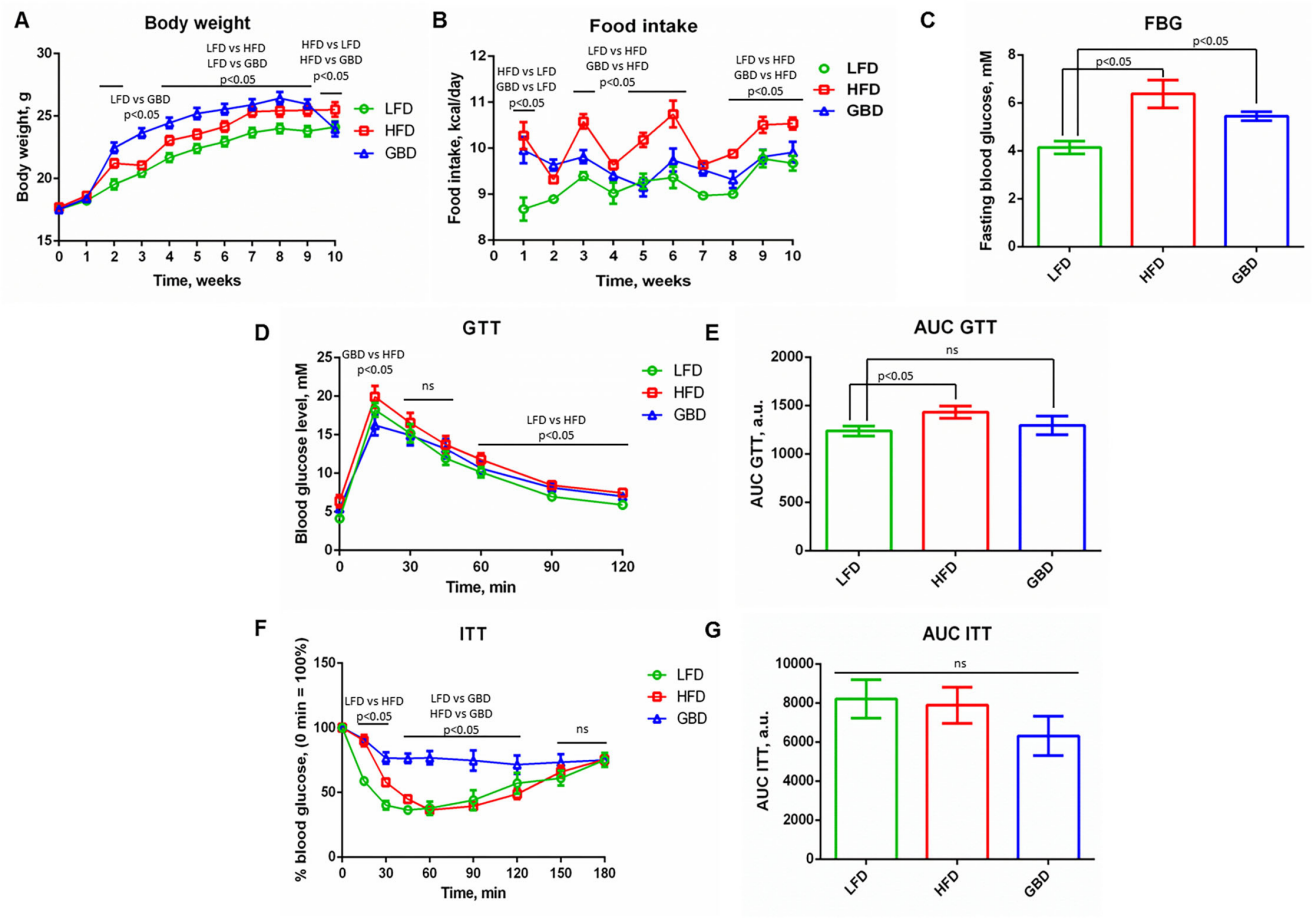
Metabolic parameters of LFD-, HFD-, and GBD-fed mice after 10 weeks of feeding, pre-surgery state. **(A)** Development of body weight; **(B)** Food intake level; **(C)** Fasting blood glucose level; **(D)** Kinetics of blood glucose level during GTT; **(F)** Kinetics of blood glucose level during ITT. The area under the curve (AUC) of GTT and ITT are shown in **(E,G)**, respectively. LFD, low fat diet; HFD, high fat diet; GBD, grain based diet; FBG, fasting blood glucose; GTT, glucose tolerance test; ITT, insulin tolerance test. Data are represented as mean ± SEM, Kruskel-Wallis test with *post-hoc* Dunn's test, significance threshold *p* < 0.05.

Body weight was significantly higher in HFD and GBD groups from the 4th to the 9th weeks of feeding compared with LFD-fed mice. Moreover, GBD-fed mice had higher body weight in comparison with HFD, despite a tendency to increase food intake only in the HFD group (Figures 1A,B). After 10 weeks of experimental feeding, animals in both HFD and GBD displayed a significantly increased FBG level in comparison with LFD-fed mice; the difference between HFD and GBD groups was nonsignificant (Figure 1C). Based on these results, we suggest the development of impairments in glucose metabolism not only in HFD but in GBD-fed mice.

Also we investigated the physiological response of animals on administration of glucose (GTT) or insulin (ITT) for the evaluation of systemic insulin sensitivity. GTT demonstrated elevated blood glucose 60, 90, and 120 min after glucose injection in the HFD group relative to LFD. HFD also displayed higher blood glucose level in comparison with the GBD group at 15 min of GTT. The AUC was greater in the HFD group than in LFD, indicating an impaired glucose tolerance in HFD-fed mice (Figures 1D,E). The analysis of ITT results revealed differences in curves (reduction of blood glucose level was stronger in LFD group in comparison with HFD and GBD groups at 15 and 30 min, and blood glucose level was stable and higher in GBD group in comparison with LFD and HFD groups from 45 to 120 min of ITT (Figure 1F). However, AUC ITT was not significantly different between all the three diet groups (Figure 1G). GTT and ITT data together testify to the development of impaired glucose tolerance in the HFD group at 10 week of diets and healthy metabolic phenotype in the LFD group.

### Systemic Metabolic Parameters of LFD-, HFD-, and GBD-Fed Mice Remain Similar During Skeletal Muscle Recovery and Postsurgical Care

At week 10 of feeding surgical modeling of hind limb, ischemia was carried out with consequent 3 weeks recovery. We analyzed the systemic metabolism of mice fed with different diets during postsurgical care and ischemic skeletal muscle recovery.

**Figure 2:**
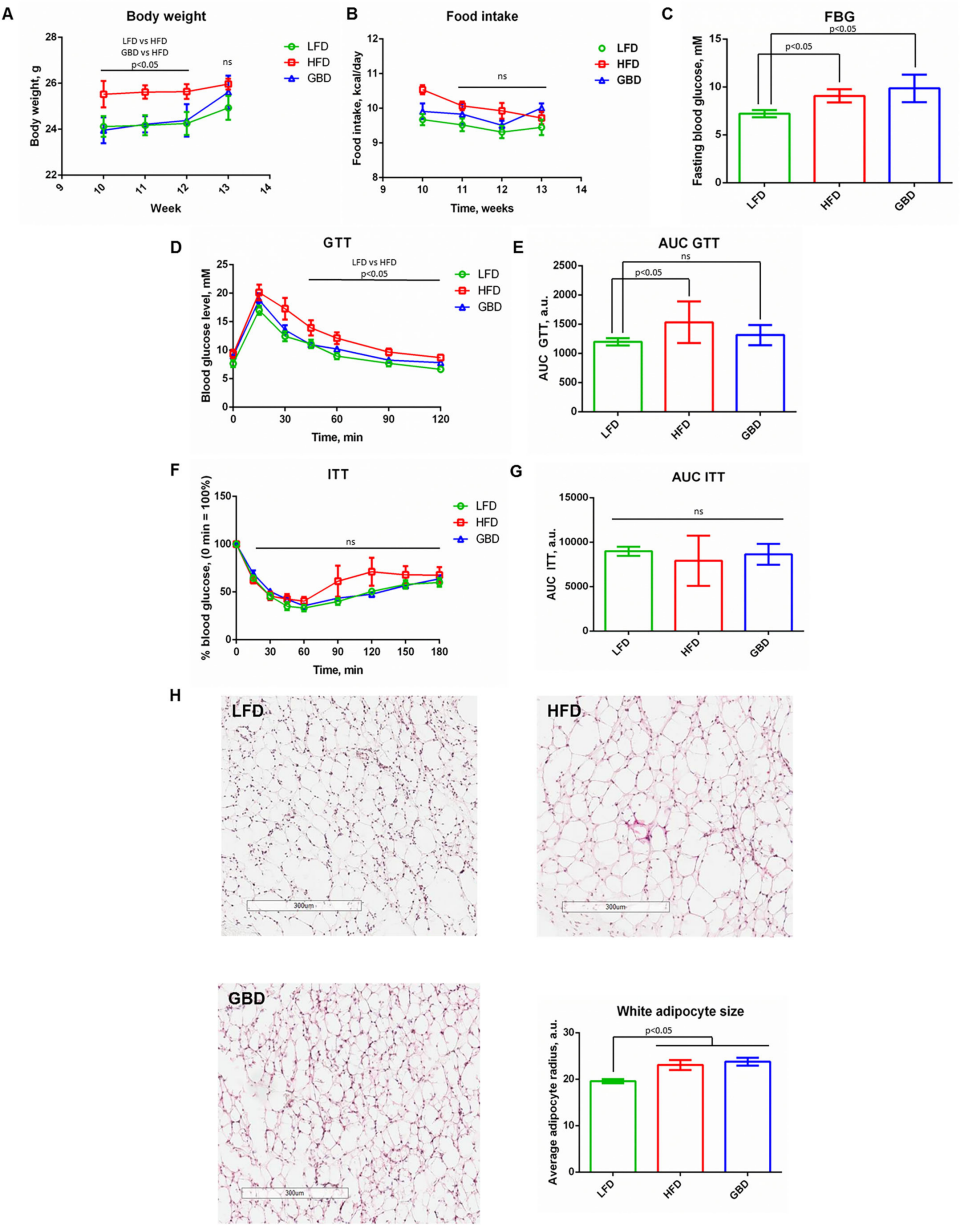
Metabolic parameters of LFD-, HFD-, and GBD-fed mice after hind limb ischemia surgical modeling at week 13, 21 days after hind limb ischemia modeling. **(A)** Body weight of experimental animals over a time course of post-surgical recovery; **(B)** Food intake level; **(C)** Fasting blood glucose level; **(D)** Kinetics of blood glucose level during GTT; **(F)** Kinetics of blood glucose level during ITT. The area under the curve (AUC) of GTT and ITT are shown in **(E,G)**, respectively. **(H)** Evaluation of the average white adipocyte size. LFD, low fat diet; HFD, high fat diet; GBD, grain based diet; FBG, fasting blood glucose; GTT, glucose tolerance test; ITT, insulin tolerance test. Data are represented as mean ± SEM, Kruskel-Wallis test with *post-hoc* Dunn's test, significance threshold *p* < 0.05. Scale-bar = 300 um.

Immediately, prior hind limb ischemia modeling and on days 7 and 14 of postsurgical care GBD-fed mice had significantly elevated body mass. Nevertheless, on day 21 of postsurgical care all mice had equal body weight and food intake (Figures 2A,B). Despite the similarity of body mass, FBG level was significantly higher in HFD and GBD groups in comparison with LFD group (Figure 2C). The analysis of insulin sensitivity at week 13 of diet showed results similar to week 10: AUC GTT was significantly higher in HFD group in comparison with LFD and GBD groups (Figures 2D,E), whereas AUC ITT was equal in all three groups (Figures 2F,G). These results suggest the development of glucose intolerance in the HFD group and relatively healthy metabolic phenotype in the LFD group. GBD-fed mice were characterized by elevated FBG level, normal AUC GTT, and normal AUC ITT (in contrast with the 10-week timepoint, Figure 1F). Moreover, the adipocyte's average size was increased in mice both on HFD and GBD in comparison with LFD-fed mice (Figure 2H). These results suggest that GBD mice do not develop glucose intolerant phenotype as HFD, but have positive energetic balance and mild metabolic alterations.

### LFD-Fed Mice Have Less Macrophage Infiltration During Post-ischemic Skeletal Muscle Recovery in Comparison With HFD- and GBD-Fed Mice

On day 21 of postsurgical care after hind limb ischemia modeling, we analyzed morphology and inflammatory macrophages infiltration of ischemic skeletal muscle.

**Figure 3:**
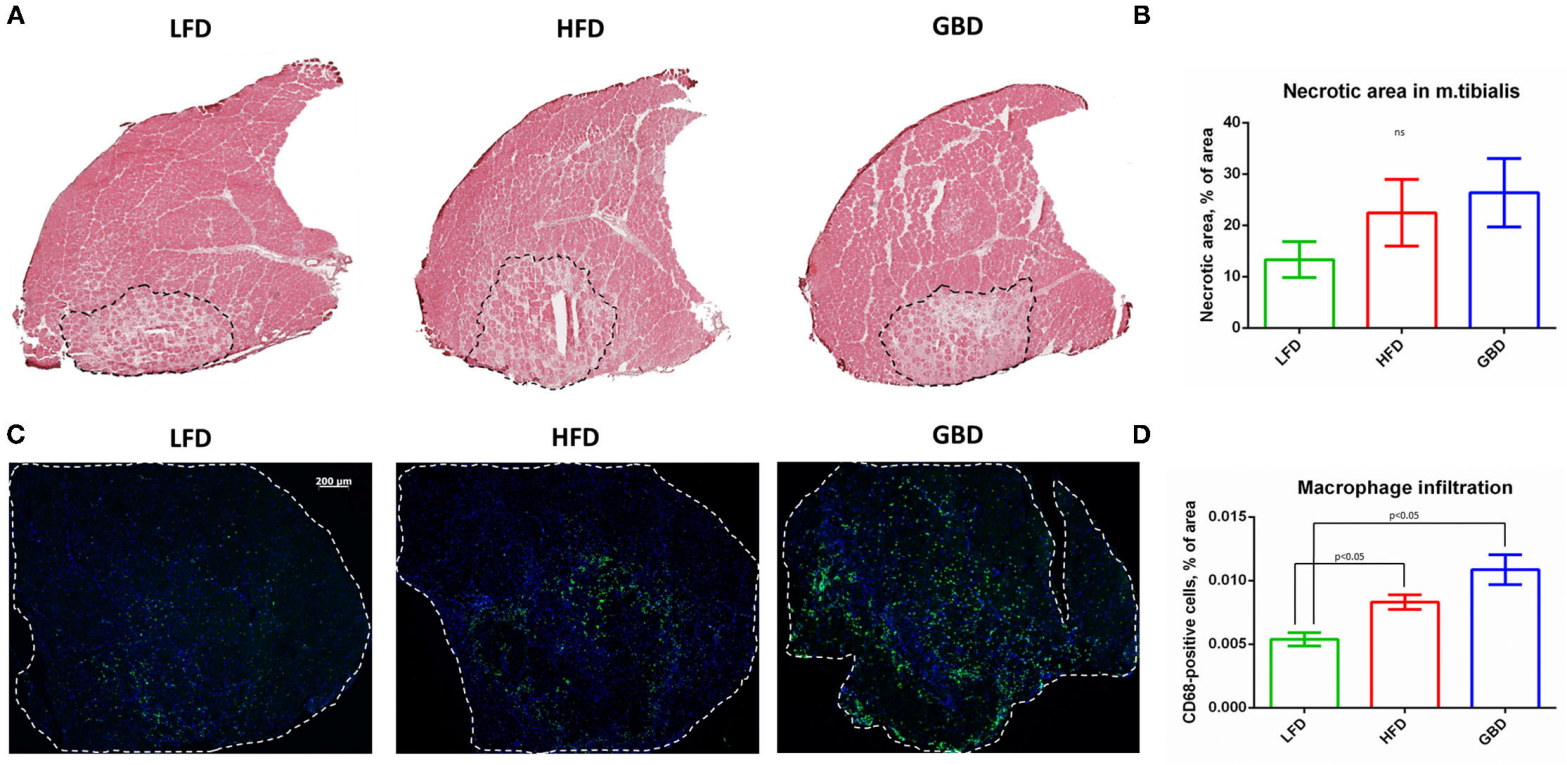
LFD-fed mice have less necrotic area and macrophage infiltration of *m. tibialis* in comparison with HFD- and GBD-fed mice. **(A)** Representative images of hematoxylin/eosin stained sections; **(B)** Percentage of necrotic area of ischemic *m. tibialis*; **(C)** Representative images of CD68-stained sections; **(D)** Percentage of macrophage infiltration of ischemic *m. tibialis*; *m. tibialis* is delineated with white dashed line. CD68, cluster of differentiation type 68. Data are represented as mean ± SEM, Kruskel–Wallis test with *post-hoc* Dunn's test, significance threshold *p* < 0.05.

Necrotic area in *m. tibialis*, an important reporter of post-ischemic recovery, was not significantly changed between dietary groups. However, in the LFD group we found a tendency to reduce necrotic area in comparison with other dietary groups (Figure 3A). Regarding macrophage infiltration, the LFD group had significantly lower CD68-macrophage content in comparison with either HFD or GBD groups (Figure 3B). In summary, LFD-fed mice exhibited more hopeful results in necrotic area and macrophage infiltration in comparison with HFD and GBD groups, which had a tendency to higher necrosis and inflammation in *m. tibialis* during post-ischemic recovery.

### GBD-Fed Mice Demonstrate Impaired Arteriogenesis and Tendency to Decreased Post-ischemic Blood Flow Recovery

Angiogenesis plays an important role in post-ischemic recovery of skeletal muscle allowing supply of nutrients and soluble factors for vessel growth.

**Figure 4:**
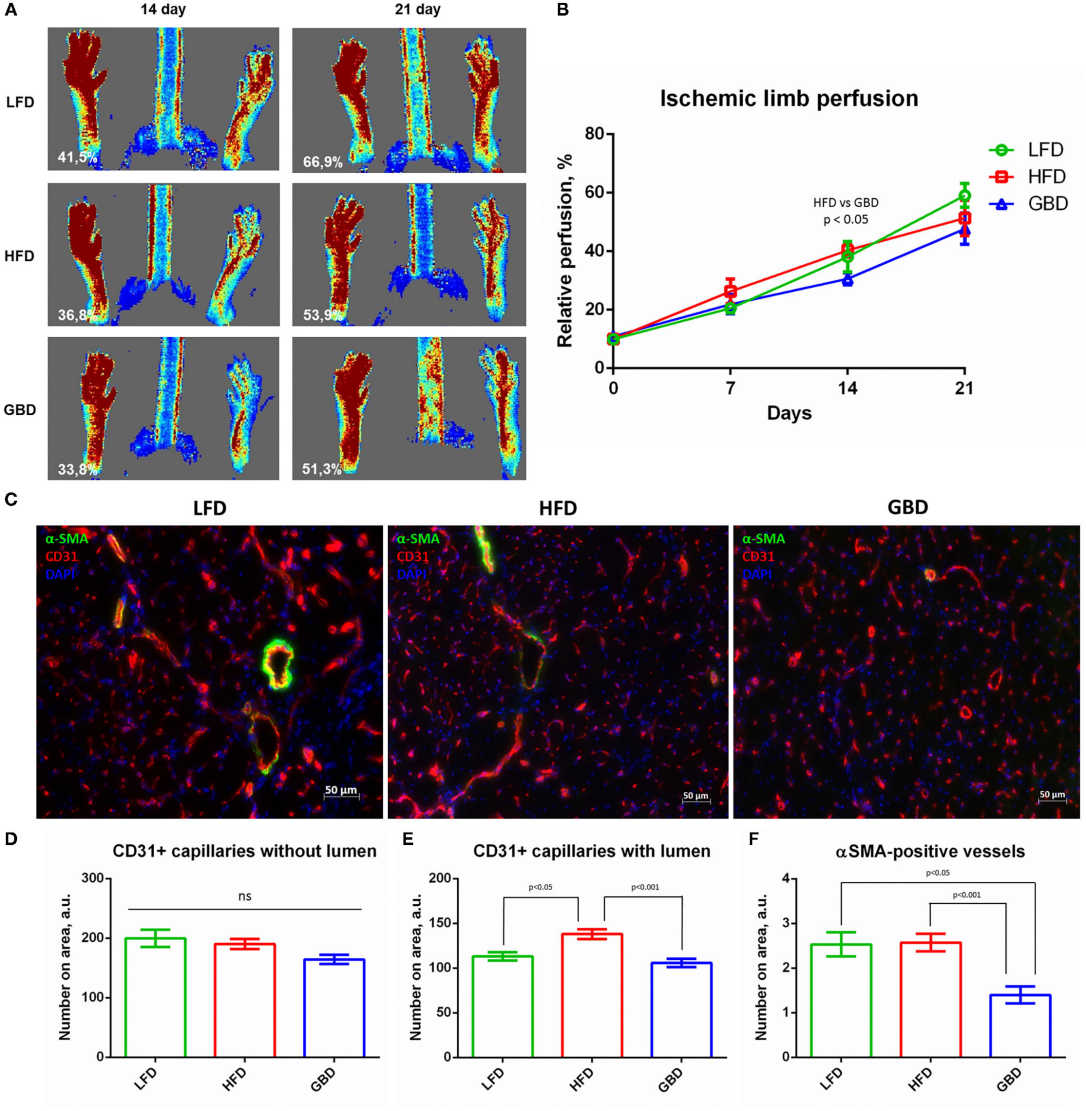
GBD-fed mice have impaired blood flow recovery in ischemic limb *via* impairment of □-SMA-positive vessels formation in *m. tibialis*. **(A)** Representative Laser Doppler scanning images of subcutaneous blood flow in mice from experimental groups obtained on day 21 after surgical ischemia induction; **(B)** Limb perfusion graph indicating relative perfusion values; **(C)** Representative image of m.tibialis sections stained for CD31, αSMA, and DAPI; graphical presentation of blood vessel density analysis with average group values per field of view: CD31+ capillaries with lumen **(D)**, CD31+ capillaries without lumen **(E)**, and αSMA-positive vessels **(F)**. CD31, cluster of differentiation type 31; αSMA, alpha smooth muscle actin; DAPI, 4′,6-diamidino-2-phenylindole. Data are represented as mean ± SEM, Kruskel–Wallis test with *post-hoc* Dunn's test, significance threshold *p* < 0.05. Scale-bar = 50 μm.

Laser Doppler scanning analysis has shown that on day 7 after surgery subcutaneous blood flow was equal in all the three groups. On day 14 after surgery GBD-fed mice had significantly decreased recovery of subcutaneous blood flow in comparison with HFD mice. On day 21 after surgery, GBD-fed mice also had the lowest rate of subcutaneous blood flow in comparison with HFD and LFD groups, but this difference was statistically insignificant (Figures 4A,B). We can tightly speculate that GBD-fed mice have a trend to impaired recovery of blood flow in ischemic limb. Histological analysis of angiogenesis in ischemic *m. tibialis* demonstrated statistically equal grade of capillaries without lumen (Figures 4C,D), enhanced formation of capillaries with lumen in HFD-fed mice ischemic *m. tibialis* (≈15% enhancing; Figures 4C,E), and dramatical suppression of arteriogenesis in GBD-fed mice (two-fold decrease; Figures 4C,F). In this light, we can suggest that tendencies to impaired recovery of both blood flow and necrotic area, likewise high macrophage infiltration level, can be caused by disturbances in arteriogenesis in ischemic skeletal muscle.

### Downregulated Ischemic Hind Limb Recovery Under GBD Concatenates With Reduced Glucose Uptake and GLUT4 Expression in Skeletal Muscle

To provide insight into possible mechanisms of impaired arteriogenesis, we analyzed the ischemic skeletal muscle energy supply with carbohydrates under different dietary interventions.

**Figure 5:**
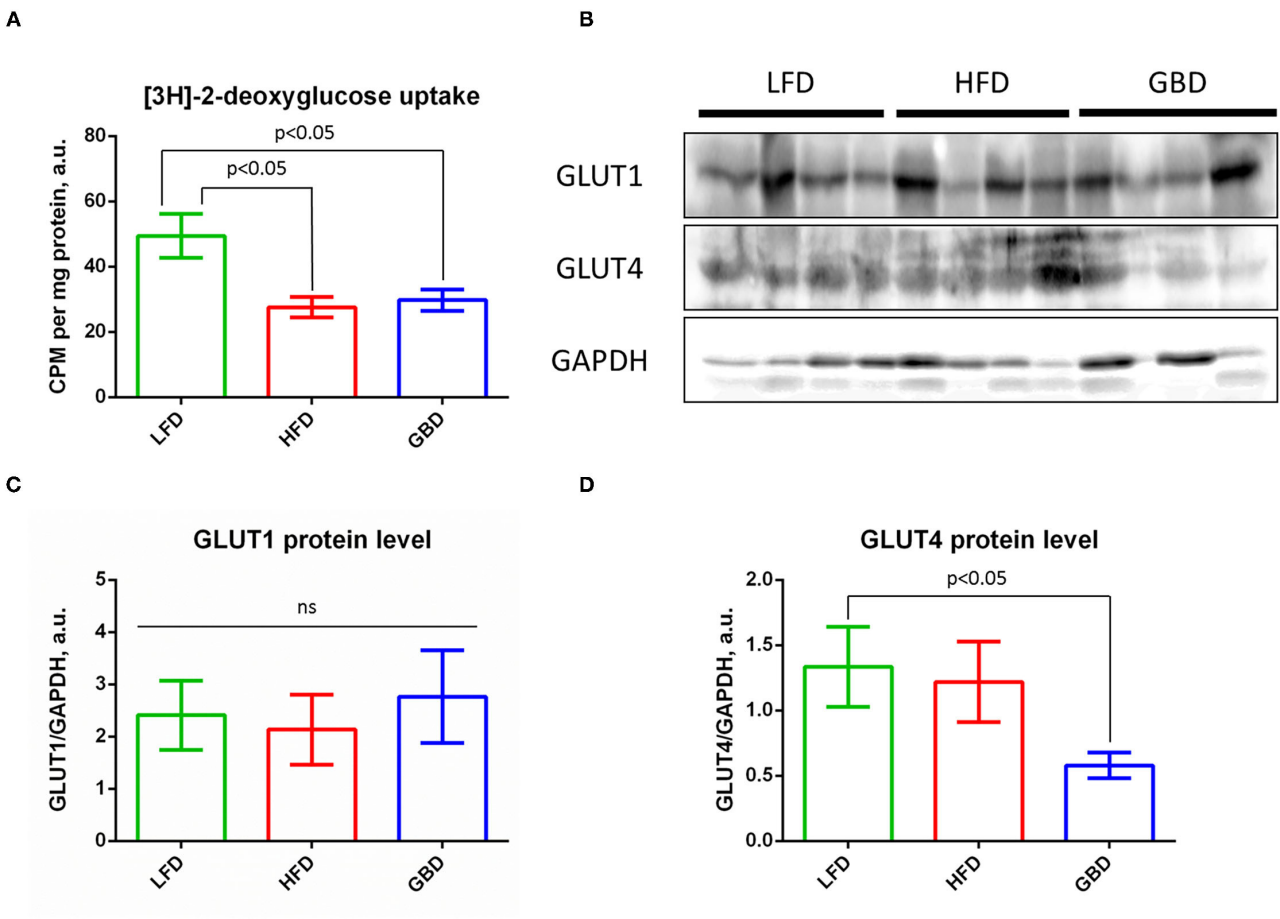
GBD-fed mice have impaired basal glucose uptake in complex with suppressed GLUT4 expression. **(A)** Statistical analysis of insulin-stimulated [3H]-2-deoxyglucose uptake; **(B)** Representative Western blots of GLUT1 and GLUT4 expression, GAPDH, loading control; **(C)** Statistical analysis of GLUT1 expression, GAPDH normalized; **(D)** Statistical analysis of GLUT4 expression, GAPDH normalized. GLUT1, glucose transporter type 1; GLUT4, glucose transporter type 4; GAPDH, glyceraldehyde phosphate dehydrogenase. Data are represented as mean ± SEM, Kruskel–Wallis test with *post-hoc* Dunn's test, significance threshold *p* < 0.05.

High-fat diet or GBD feeding leads to suppression of glucose uptake by skeletal muscle (Figure 5A). This result is consistent with impaired blood flow recovery and arteriogenesis in ischemic hind limbs of GBD-fed mice. Analysis of the main glucose transporters expression showed equal level of GLUT1 expression (Figures 5B,C), but decreased level of GLUT4 expression in skeletal muscle of GBD-fed mice (Figures 5B,D). These data show the role of diet and insulin sensitivity in energy supply of regenerative processes.

## Discussion

Whole grain and low-fat diets offer benefits during different metabolic complications in comparison with high fat diets. In our work, HFD-fed mice had impaired glucose tolerance and adipocyte hypertrophy in contrast to LFD. GBD-fed mice had intermediate metabolic phenotype, demonstrating impaired FBG and adipocytes hypertrophy together with normal glucose tolerance and insulin sensitivity. These data suggest that GBD induces the initial stage of metabolic abnormalities. GBD composition differs from LFD in simple sugars, oligo-, and polysaccharides content, and in the presence of phytohormones leading to development of a distinct metabolic phenotype (43, 44). Our observations are inconsistent with studies, reporting improvement of insulin sensitivity on GBD both in animals (45, 46) and humans (47, 48). However, other investigations demonstrated no influence of whole grain intake on insulin sensitivity (49, 50). Thus, further we analyze post-ischemic recovery under normal metabolic conditions (LFD), initial metabolic complications (GBD), and impaired glucose tolerance (HFD).

Despite differences in severity of metabolic impairments, we found a similar degree of the *m. tibialis* necrosis in all groups on day 21 after ischemia. However, muscle recovery tended to decline in HFD- and GBD-fed mice in comparison with LFD. Furthermore, our findings indicate increased macrophage infiltration of *m. tibialis* in HFD- and GBD-fed mice. This is in accordance with previous reports in the HFD model and supports the idea that elevated inflammation promotes necrosis development (Figure 3) (16, 51). However, GBD was reported to suppress inflammation both in the experimental and clinical studies in contrast to our results (37–40). Probably, GBD can regulate inflammation and promote muscle regeneration by microbiome-mediated mechanisms (40).

Restoration of blood flow after ischemia is essential for tissue regeneration and to regain its functional activity. Ischemic limb perfusion was partially restored after day 21 of recovery period in all the experimental groups. However, in mice fed GBD blood flow was the lowest. Further, we found that the number of capillaries was equal in all the groups, and only the number of capillaries with lumen was increased on HFD. This observation coincides with findings that HFD can accelerate vascularization and skeletal muscle regeneration in ischemic animal model (24). In contrast, the density of vessels with smooth muscle wall was decreased on GBD, suggesting attenuation of arteriogenesis. This effect can be mediated via changes of inflammatory state on GBD, known to affect arteriogenesis (52–55).

Impaired skeletal muscle regeneration can be the consequence of deregulated metabolism and energetic substrates intake. We found that *m. tibialis* explants of HFD- and GBD-fed mice had suppressed glucose uptake in comparison with the LFD group. We suggest that HFD-fed mice probably use lipids for energy supply of regenerative processes and do not need high glucose uptake rate. Indeed, suppression of basal glucose uptake in muscles of the HFD group was not associated with impaired angiogenesis/arteriogenesis or muscle regeneration. In contrast, downregulation of glucose uptake in muscle of GBD-fed mice was accompanied with necrosis and decreased blood flow. Probably, insufficient nutritional supply of satellite cells and smooth muscle progenitor cells on GBD leads to an impairment of muscle regeneration and arteriogenesis processes. In normal conditions, both skeletal muscle recovery and angiogenesis stimulate GLUT4 expression for increase of energy intake in damaged cells (56, 57). However, GLUT4 expression was decreased on GBD, which could be the consequence of excessive blood glucose level (58, 59). Therefore, we suggest that a high dietary carbohydrate level on GBD leads to hyperglycemia, reduction of GLUT4 expression, and decrease of energy supply of regenerative processes.

In summary, we suggest that different dietary backgrounds can critically affect the processes of angiogenesis and muscle recovery after ischemia. LFD background supports more effective muscle regeneration in comparison with HFD and, especially, GBD. According to our results GBD cannot be used as a relevant control instead of LFD in works of regeneration and obesity.

## Conclusion

We conclude that dietary background determines the rate of post-ischemic regeneration including angiogenesis, skeletal muscle recovery, and metabolic activity. The most effective regeneration is supported by LFD, whereas the lowest rate of regeneration occurs on GBD. It can be related to impaired glucose uptake and GLUT4 expression on GBD leading to insufficient energy supply. The obtained results raise a question about the applicability of grain-based dietary interventions during PAD and post-ischemic recovery.

## Data Availability Statement

The raw data supporting the conclusions of this article will be made available by the authors, without undue reservation.

## Ethics Statement

The animal experiment was performed in accordance with the EU Directive 2010/63/EU for animal experiments and approved by Institutional Ethics Board for Animal Care (National Medical Research Center for Cardiology, permit #385.06.2009).

## Author Contributions

IS, MB, and SM collected, analyzed, interpreted the data, and wrote and reviewed the manuscript. EM and ER collected and analyzed the data. MM reviewed the manuscript. YP designed and supervised the study and reviewed the manuscript. All authors have read and approved the final version of the manuscript.

## Funding

This work was supported by Russian Science Foundation grant #20-45-08003 (in part of diets modeling, analysis of animal's systemic metabolism, and *ex vivo* glucose metabolism assay) and Russian Foundation of Basic Research grant #20-015-00181 (in part of hind limb ischemia modeling, evaluation of blood flow recovery, and angiogenesis in ischemic skeletal muscle).

## Conflict of Interest

The authors declare that the research was conducted in the absence of any commercial or financial relationships that could be construed as a potential conflict of interest.

## Publisher's Note

All claims expressed in this article are solely those of the authors and do not necessarily represent those of their affiliated organizations, or those of the publisher, the editors and the reviewers. Any product that may be evaluated in this article, or claim that may be made by its manufacturer, is not guaranteed or endorsed by the publisher.

## Supplementary Material

Supplementary Figure 1 Graphical representation of study design. LFD, low fat diet; HFD, high fat diet; GBD, grain-based diet; FBG, fasting blood glucose; GTT, glucose tolerance test; ITT, insulin tolerance test.

Supplementary Figure 2 Histological analysis of insulin-sensitive tissues. **(A)** Representative images of brown adipose tissue morphology under experimental diets, hematoxylin/eosin staining; **(B)** The estimation of brown adipocytes average size under experimental diets; **(C)** Representative images of liver morphology under experimental diets, OilRedO/hematoxylin/eosin staining; **(D)** The estimation of lipid accumulation under experimental diets; E, representative images of *m. tibialis* morphology under experimental diets. LFD, low fat diet; HFD, high fat diet; GBD, grain-based diet; H&E, hematoxylin/eosin staining. Data are represented as mean ± SEM, Kruskel-Wallis test with *post-hoc* Dunn's test, significance threshold *p* < 0.05. Scale-bar = 100 um.

Supplementary Table 1 Compositions of LFD, HFD, and GBD which have been used in a present study.

Supplementary Table 2 Compound-based compositions of LFD, HFD, and GBD which have been used in a present study.

## Abbreviations

AUC: area under curve
AMP: adenosine monophosphate
BSA: bovine serum albumin
CD31, 68: cluster of differentiation type 31, 68
DMEM: Dulbecco's modified Eagle medium
FBG: fasting blood glucose
GBD: grain-based diet
GTT: glucose tolerance test
GLUT1, 4: glucose transporters type 1, 4
GAPDH: glyceraldehyde phosphate dehydrogenase
HFD: high-fat diet
ITT: insulin tolerance test
LFD: low-fat diet
OXPHOS: oxidative phosphorylation
PAD: peripheral artery disease
PTEN: phosphatase and tensin homolog
RIPA: radioimmunoprecipitation assay buffer
αSMA: α-smooth muscle actin
SDS: sodium dodecyl sulfate
SEM: standard error mean
T2DM: type 2 diabetes mellitus.

## References

[1] Chalvon-Demersay T Blachier F Tomé D Blais A. Animal models for the study of the relationships between diet and obesity: a focus on dietary protein and estrogen deficiency. Front Nutr. (2017) 4:5. 10.3389/fnut.2017.00005 28373974 PMC5357654

[2] Gardner AW Bright BC Ort KA Montgomery PS. Dietary intake of subjects with peripheral artery disease and claudication. Angiology. (2011) 62:270–5. 10.1177/0003319710384395 21406424 PMC4141709

[3] Brostow DP Hirsch AT Collins TC Kurzer MS. The role of nutrition and body composition in peripheral arterial disease. Nat Rev Cardiol. (2012) 9:634–43. 10.1038/nrcardio.2012.117 22922595 PMC4535926

[4] Gardner AW Montgomery PS Wang M Shen B Casanegra A Silva-Palacios F. Diet is associated with ankle-brachial index, inflammation, and ambulation in patients with intermittent claudication. J Vasc Surg. (2020) 72:1375–84. 10.1016/j.jvs.2019.12.038 32122735 PMC7483568

[5] Malik VS Willet WC Hu FB. Nearly a decade on — trends, risk factors and policy implications in global obesity. Nat Rev Endocrinol. (2020) 16:615–6. 10.1038/s41574-020-00411-y 32873971 PMC7461756

[6] Zhang P Sun X Jin H Zhang FL Guo ZN Yang Y. Association between obesity type and common vascular and metabolic diseases: a cross-sectional study. Front Endocrinol. (2020) 10:900. 10.3389/fendo.2019.00900 31998234 PMC6962099

[7] Powell-Wiley TM Poirier P Burke LE Després JP Gordon-Larsen P Lavie CJ. Obesity and cardiovascular disease: a scientific statement from the American heart association. Circulation. (2021) 143:e984–1010. 10.1161/CIR.0000000000000973 33882682 PMC8493650

[8] Beckman JA Creager MA. Vascular complications of diabetes. Circ Res. (2016) 118:1771–85. 10.1161/CIRCRESAHA.115.306884 27230641

[9] Barnes JA Eid MA Creager MA Goodney PP. Epidemiology and risk of amputation in patients with diabetes mellitus and peripheral artery disease. Ather Thromb Vasc Biol. (2020) 40:1808–17. 10.1161/ATVBAHA.120.314595 32580632 PMC7377955

[10] Hazarika S Dokun AO Li Y Popel AS Kontos CD Annex BH. Impaired angiogenesis after hindlimb ischemia in type 2 diabetes mellitus: differential regulation of vascular endothelial growth factor receptor 1 and soluble vascular endothelial growth factor receptor 1. Circ Res. (2007) 101:948–56. 10.1161/CIRCRESAHA.107.160630 17823371

[11] Sodha NR Boodhwani M Clements RT Xu SH Khabbaz KR Sellke FW. Increased antiangiogenic protein expression in the skeletal muscle of diabetic swine and patients. Arch Surg. (2008) 143:463–70. 10.1001/archsurg.143.5.463 18490555 PMC2617725

[12] Kalogeris T Baines CP Krenz M Korthuis RJ. Cell biology of ischemia/reperfusion injury. Int Rev Cell Mol Biol. (2012) 298:229–317. 10.1016/B978-0-12-394309-5.00006-7 22878108 PMC3904795

[13] Hu Z Wang H Lee IH Modi S Wang X Du J. PTEN inhibition improves muscle regeneration in mice fed a high-fat diet. Diabetes. (2010) 59:1312–20. 10.2337/db09-1155 20200318 PMC2874691

[14] Fu X Zhu M Zhang S Foretz M Viollet B Du M. Obesity impairs skeletal muscle regeneration through inhibition of AMPK. Diabetes. (2016) 65:188–200. 10.2337/db15-0647 26384382 PMC4686944

[15] Jin H Oh HJ Kim J Lee KP Han X Lee OH. Effects of ecklonia stolonifera extract on the obesity and skeletal muscle regeneration in high-fat diet-fed mice. J Funct Food. (2021) 82:104511. 10.1016/j.jff.2021.104511

[16] Albadawi H Oklu R Cormier NR O'Keefe RM Heaton JT Kobler JB. Hind limb ischemia-reperfusion injury in diet-induced obese mice. J Surg Res. (2014) 190:683–91. 10.1016/j.jss.2014.01.020 24655666 PMC4096585

[17] Molnar J Yu S Mzhavia N Pau C Chereshnev I Dansky HM. Diabetes induces endothelial dysfunction but does not increase neointimal formation in high-fat diet fed C57BL/6J mice. Circ. Res. (2005) 96:1178–84. 10.1161/01.RES.0000168634.74330.ed 15879311

[18] Costa RR Villela NR Souza M Boa BC Cyrino FZ Silva SV. High fat diet induces central obesity, insulin resistance and microvascular dysfunction in hamsters. Microvasc Res. (2011) 82:416–22. 10.1016/j.mvr.2011.08.007 21889944

[19] Petersen MC Shulman GI. Mechanisms of insulin action and insulin resistance. Physiol Rev. (2018) 98:2133–223. 10.1152/physrev.00063.2017 30067154 PMC6170977

[20] Rydén M Andersson DP Bernard S Spalding K Arner P. Adipocyte triglyceride turnover and lipolysis in lean and overweight subjects. J Lipid Res. (2013) 54:2909–13. 10.1194/jlr.M040345 23899442 PMC3770103

[21] Arner P Ryden M. Fatty acids, obesity and insulin resistance. Obes Facts. (2015) 8:147–55. 10.1159/000381224 25895754 PMC5644864

[22] Aday AW Everett BM. Dyslipidemia profiles in patients with peripheral artery disease. Curr Cardiol Rep. (2019) 21:42. 10.1007/s11886-019-1129-5 31011836 PMC7220794

[23] Chazaud B. Inflammation and skeletal muscle regeneration: leave it to the macrophages!. Trends Immunol. (2020) 41:481–92. 10.1016/j.it.2020.04.006 32362490

[24] Nwadozi E Rudnicki M De Ciantis 1 Milkovich S Pulbere A Roudier E. High-fat diet pre-conditioning improves microvascular remodelling during regeneration of ischaemic mouse skeletal muscle. Acta Physiol. (2020) 229:e13449. 10.1111/apha.13449 32012450

[25] Kulezic A Bergwall S Fatemi S Sonestedt E Zarrouk M Gottsäter A. Healthy diet and fiber intake are associated with decreased risk of incident symptomatic peripheral artery disease - a prospective cohort study. Vasc Med. (2019) 24:511–8. 10.1177/1358863X19867393 31431146

[26] Delaney CL Smale MK Miller MD. Nutritional considerations for peripheral arterial disease: a narrative review. Nutrients. (2019) 11:1219. 10.3390/nu11061219 31146408 PMC6627356

[27] Kopf JC Suhr MJ Clarke J Eyun SI Riethoven JM Ramer-Tait AE. Role of whole grains versus fruits and vegetables in reducing subclinical inflammation and promoting gastrointestinal health in individuals affected by overweight and obesity: a randomized controlled trial. Nutr J. (2018) 17:72. 10.1186/s12937-018-0381-7 30060746 PMC6066923

[28] Maki KC Palacios OM Koecher K Sawicki CM Livingston KA Bell M. The relationship between whole grain intake and body weight: results of meta-analyses of observational studies and randomized controlled trials. Nutrients. (2019) 11:1245. 10.3390/nu11061245 31159235 PMC6627338

[29] Hu Y Ding M Sampson L Willett WC Manson JE Wang M. Intake of whole grain foods and risk of type 2 diabetes: results from three prospective cohort studies. BMJ. (2020) 370:m2206. 10.1136/bmj.m2206 32641435 PMC7341349

[30] Klempel MC Kroeger CM Norkeviciute E Goslawski M Phillips SA Varady KA. Benefit of a low-fat over high-fat diet on vascular health during alternate day fasting. Nutr Diabetes. (2013) 3:e71. 10.1038/nutd.2013.14 23712283 PMC3671747

[31] Nosova EV Conte MS Grenon SM. Advancing beyond the “heart-healthy diet” for peripheral arterial disease. J Vasc Surg. (2015) 61:265–74. 10.1016/j.jvs.2014.10.022 25534981 PMC4275620

[32] Holecek T. Nutritional modulation of liver regeneration by carbohydrates, lipids, and amino acids: a review. Nutrition. (1999) 15:784–8. 10.1016/S0899-9007(99)00158-6 10501293

[33] Dinu M Whittaker A Pagliai G Giangrandi I Colombini B Gori AM. A khorasan wheat-based replacement diet improves risk profile of patients with nonalcoholic fatty liver disease (NAFLD): a randomized clinical trial. J Am Coll Nutr. (2018) 37:508–14. 10.1080/07315724.2018.1445047 29652567

[34] Giusti L Gabriele M Penno G Garofolo M Longo V Del Prato S. A fermented whole grain prevents lipopolysaccharides-induced dysfunction in human endothelial progenitor cells. Oxid Med Cell Longev. (2017) 2017:1026268. 10.1155/2017/1026268 28386305 PMC5366772

[35] Zhou JY Zhang SW Lin HL Gao CQ Yan HC Wang XQ. Hydrolyzed wheat gluten alleviates deoxynivalenol-induced intestinal injury by promoting intestinal stem cell proliferation and differentiation via upregulation of Wnt/β-catenin signaling in mice. Food Chem Toxicol. (2019) 131:110579. 10.1016/j.fct.2019.110579 31202940

[36] Wang Z Dabrosin C Yin X Fuster MM Arreola A Rathmell WK. Broad targeting of angiogenesis for cancer prevention and therapy. Semin Cancer Biol. (2015) 35 (Suppl):S224–43. 10.1016/j.semcancer.2015.01.001 25600295 PMC4737670

[37] Zamaratskaia G Omar NAM Brunius C Hallmans G Johansson JE Andersson SO. Consumption of whole grain/bran rye instead of refined wheat decrease concentrations of TNF-R2, e-selectin, and endostatin in an exploratory study in men with prostate cancer. Clin Nutr. (2020) 39:P159–65. 10.1016/j.clnu.2019.01.007 30685298

[38] Okita K Iwahashi H Kozawa J Okauchi Y Funahashi T Imagawa A. Usefulness of the insulin tolerance test in patients with type 2 diabetes receiving insulin therapy. J Diabetes Investig. (2014) 5:305–12. 10.1111/jdi.12143 24843779 PMC4020335

[39] Niiyama H Huang NF Rollins MD Cooke JP. Murine model of hindlimb ischemia. J Vis Exp. (2009) 23:1035. 10.3791/1035 19229179 PMC2763292

[40] Yu J Dardik A. A murine model of hind limb ischemia to study angiogenesis and arteriogenesis. Methods Mol Biol. (2018) 1717:135–43. 10.1007/978-1-4939-7526-6_11 29468589 PMC5993192

[41] Boldyreva M Makarevich P Rafieva L Beloglazova I Dergilev K Kostrov S. Delivery of nerve growth factor (NGF) gene via recombinant plasmid vector induces angiogenesis in murine ischemic hind limb. Genes Cells. (2014) 9:81–7.

[42] Gagnon RC Peterson JJ. Estimation of confidence intervals for area under the curve from destructively obtained pharmacokinetic data. J Pharmacokinet Pharmacodyn. (1998) 26:87–102. 9773394 10.1023/a:1023228925137

[43] Ricci M. Laboratory animal control diets: very important, often neglected. Lab Anim. (2015) 44:240–1. 10.1038/laban.786

[44] Pellizzon MA Ricci M. Choice of laboratory rodent diet may confound data interpretation and reproducibility. Curr.Dev.Nutr. (2020) 4:nzaa031. 10.1093/cdn/nzaa031 32258990 PMC7103427

[45] Zhou AL Hergert N Rompato G Lefevre M. Whole grain oats improve insulin sensitivity and plasma cholesterol profile and modify gut microbiota composition in C57BL/6J mice. J Nutr. (2015) 145:222–30. 10.3945/jn.114.199778 25644341

[46] Teradal D Joshi N Aladakatti RH. Therapeutic evaluation of grain based functional food formulation in a geriatric animal model. J Food Sci Technol. (2017) 54:2789–96. 10.1007/s13197-017-2715-4 28928518 PMC5583108

[47] Liese AD Roach AK Sparks KC Marquart L D'Agostino RB Mayer-Davis EJ. Whole-grain intake and insulin sensitivity: the Insulin resistance atherosclerosis study. Am J Clin Nutr. (2003) 78:965–71. 10.1093/ajcn/78.5.965 14594783

[48] Tester JM Stiers KB Garber A Leung CW. Whole grain intake and impaired fasting glucose in adolescents, national health and nutrition examination survey, 2005-2014. Prev Chronic. Dis. (2020) 17:190439. 10.5888/pcd17.190439 33092687 PMC7587298

[49] Ampatzoglou A Atwal KK Maidens CM Williams CL Ross AB Thielecke F. Increased whole grain consumption does not affect blood biochemistry, body composition, or gut microbiology in healthy, low-habitual whole grain consumers. J Nutr. (2015) 145:215–21. 10.3945/jn.114.202176 25644340

[50] van Trijp MPH Schutte S Esser D Wopereis S Hoevenaars FPM Hooiveld J. Minor changes in the composition and function of the gut microbiota during a 12-week whole grain wheat or refined wheat intervention correlate with liver fat in overweight and obese adults. J Nutr. (2021) 151:491–502. 10.1093/jn/nxaa312 33188417 PMC7948209

[51] Chen L Wang L Li Y Wuang L Liu Y Pang N. Transplantation of normal adipose tissue improves blood flow and reduces inflammation in high fat fed mice with hindlimb ischemia. Front Physiol. (2018) 9:197. 10.3389/fphys.2018.00197 29568274 PMC5852102

[52] Vanegas SM Meydani M Barnett JB Goldin B Kane A Rasmussen H. Substituting whole grains for refined grains in a 6-wk randomized trial has a modest effect on gut microbiota and immune and inflammatory markers of healthy adults. Am J Clin Nutr. (2017) 105:635–50. 10.3945/ajcn.116.146928 28179226 PMC5320415

[53] Xu Y Wan Q Feng J Du L Li K Zhou Y. Whole grain diet reduces systemic inflammation. Medicine. (2018) 97:e12995. 10.1097/MD.0000000000012995 30412134 PMC6221555

[54] Tomay F Marinelli A Leoni V Caccia C Matros A Mock HP. Purple corn extract induces long-lasting reprogramming and M2 phenotypic switch of adipose tissue macrophages in obese mice. J Transl Med. (2019) 17:237. 10.1186/s12967-019-1972-6 31337415 PMC6651915

[55] Park H Yu S Kim W. Rice bran oil attenuates chronic inflammation by inducing M2 macrophage switching in high-fat diet-fed obese mice. Foods. (2021) 10:359. 10.3390/foods10020359 33562395 PMC7914799

[56] Kaliman P Viñals F Testar X Palacin M Zorzano A. Phosphatidylinositol 3-kinase inhibitors block differentiation of skeletal muscle cells. J Biol Chem. (1996) 271:19146–51. 10.1074/jbc.271.32.19146 8702591

[57] Zorzano A Palacín M Gumà A. Mechanisms regulating GLUT4 glucose transporter expression and glucose transport in skeletal muscle. Acta Physiol Scand. (2005) 183:43–58. 10.1111/j.1365-201X.2004.01380.x 15654919

[58] Yu Z Fantus IG. Glucose transporter 4 (Glut4) degradation is accelerated by hyperglycemia and hyperinsulinemia via a proteasome-dependent pathway. Diabetes. Abstract book of the 64th ADA Scientific Sessions. Orlando, FL: American Diabetes Association, (2004) 1261p.

[59] Xie B Chen Q Chen L Sheng Y Wang HY Chen S. The inactivation of RabGAP function of AS160 promotes lysosomal degradation of GLUT4 and causes postprandial hyperglycemia and hyperinsulinemia. Diabetes. (2016) 65:3327–40. 10.2337/db16-0416 27554475

