# Supplementary figures and images for: Grain-Based Dietary Background Impairs Restoration of Blood Flow and Skeletal Muscle During Hindlimb Ischemia in Comparison With Low-Fat and High-Fat Diets
Source: Front Nutr. 2022 Jan 10;8:809732. doi: 10.3389/fnut.2021.809732 (PMC8784406; doi:10.3389/fnut.2021.809732)

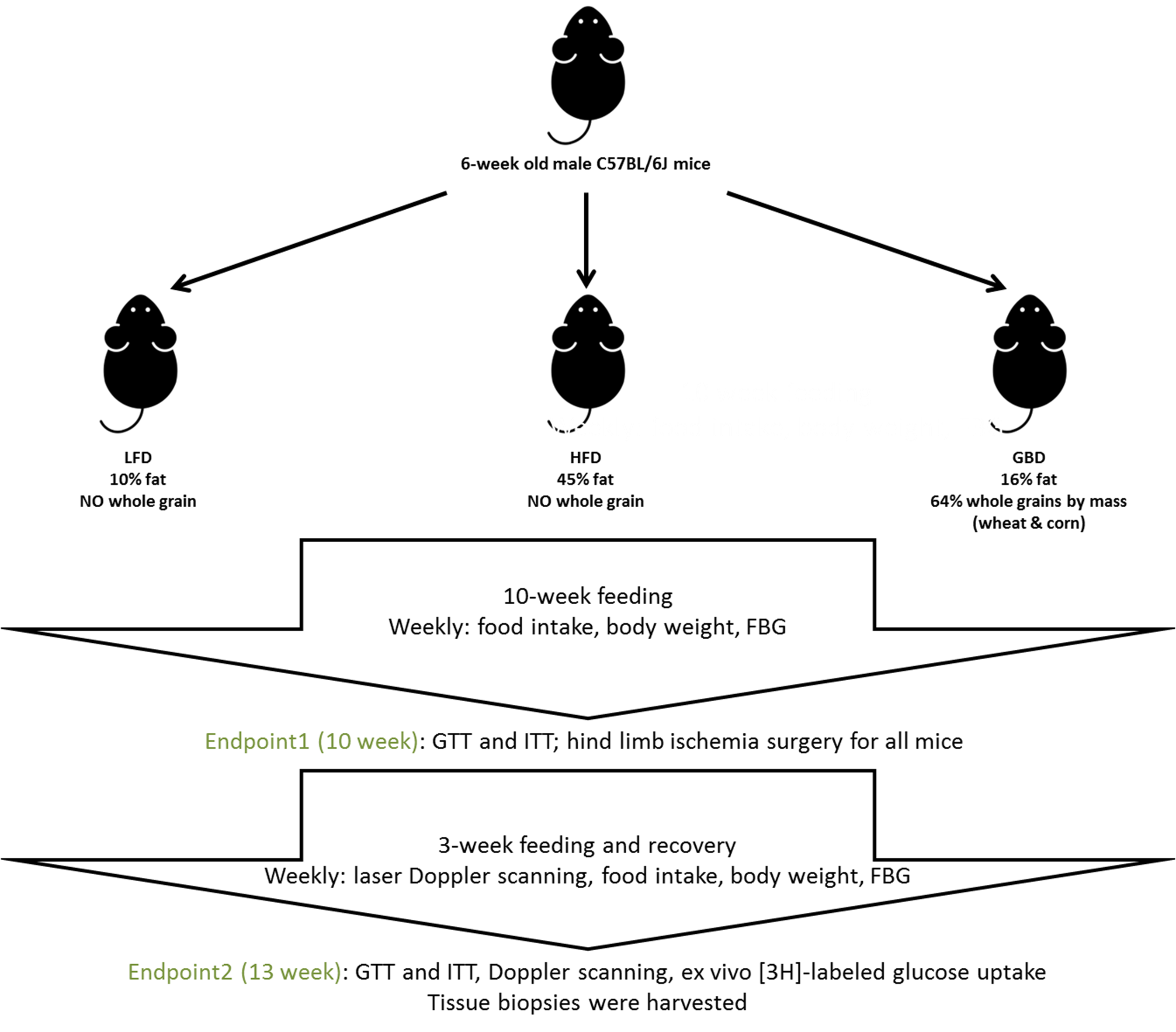

Supplement: Supplementary Figure 1 — Graphical representation of study design. LFD, low fat diet; HFD, high fat diet; GBD, grain-based diet; FBG, fasting blood glucose; GTT, glucose tolerance test; ITT, insulin tolerance test. [file Image_1.TIF]

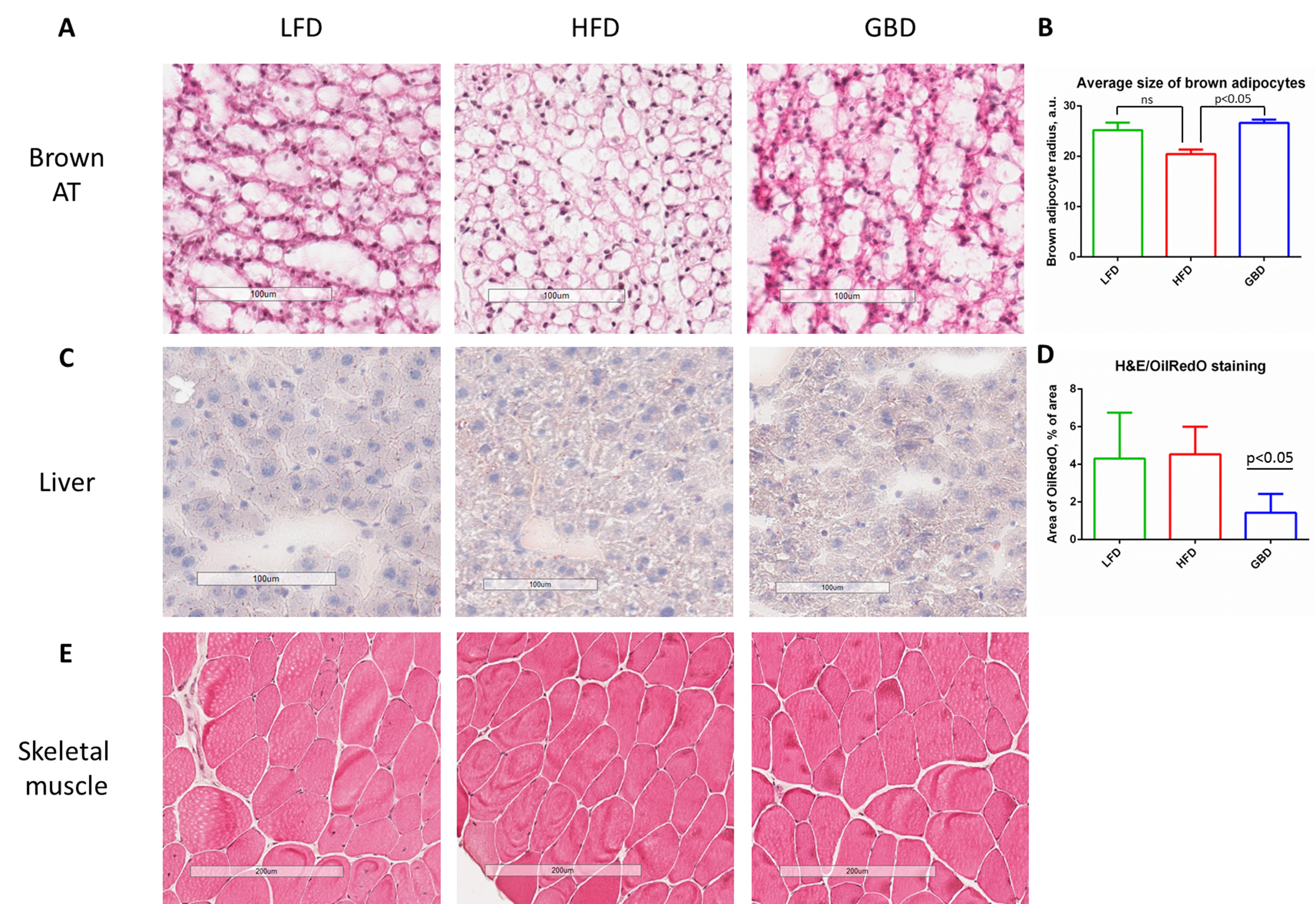

Supplement: Supplementary Figure 2 — Histological analysis of insulin-sensitive tissues. (A) Representative images of brown adipose tissue morphology under experimental diets, hematoxylin/eosin staining; (B) The estimation of brown adipocytes average size under experimental diets; (C) Representative images of liver morphology under experimental diets, OilRedO/hematoxylin/eosin staining; (D) The estimation of lipid accumulation under experimental diets; E, representative images of m. tibialis morphology under experimental diets. LFD, low fat diet; HFD, high fat diet; GBD, grain-based diet; H&E, hematoxylin/eosin staining. Data are represented as mean ± SEM, Kruskel-Wallis test with post-hoc Dunn's test, significance threshold p < 0.05. Scale-bar = 100 um. [file Image_2.TIF]
