# Supplementary material for: Grain-Based Dietary Background Impairs Restoration of Blood Flow and Skeletal Muscle During Hindlimb Ischemia in Comparison With Low-Fat and High-Fat Diets
Source: Front Nutr. 2022 Jan 10;8:809732. doi: 10.3389/fnut.2021.809732 (PMC8784406; doi:10.3389/fnut.2021.809732)
Supplement: Supplementary Table 1 — Compositions of LFD, HFD, and GBD which have been used in a present study. [file Data_Sheet_1.docx]

**Supplementary material**

Table 1. Compositions of LFD, HFD and GBD which have been used in a present study.

|  | LFD | HFD | GBD |
| --- | --- | --- | --- |
|  | Composition by % from total mass, % | | |
| Proteins | 19.22 | 23.65 | 29.9 |
| Lipids | 4.24 | 23.58 | 7.7 |
| Carbohydrates | 71.44 | 46.57 | 59.49 |
| Vitamin-mineral mixture | 5.1 | 6.2 | 2.91 |
|  | Composition by % from total kcal, % | | |
| Protein | 19.1 | 19.1 | 28 |
| Lipids | 9.6 | 43.26 | 16.35 |
| Carbohydrates | 71.3 | 37.64 | 55.65 |
|  | Whole grain by % from total mass, % | | |
| Wheat | - | - | 22 |
| Corn | - | - | 42.31 |

Table 2. Compound-based compositions of LFD, HFD and GBD which have been used in a present study

|  | LFD | HFD | GBD |
| --- | --- | --- | --- |
|  | Protein by % from total mass, % | | |
| Casein | 18.96 | 23.31 | - |
| Cystine | 0.28 | 0.35 | - |
| Soybean meal | - | - | 20 |
| Tankage | - | - | 9.2 |
|  | Carbohydrate by % from total mass, % | | |
| Corn starch | 42.86 | 8.48 | - |
| Sucrose | 16.76 | 20.61 | - |
| Lodex10 | 7.11 | 11.65 | - |
| Solka Floc | 4.73 | 5.83 | - |
|  | Lipids by % from total mass, % | | |
| Lard | 1.9 | 20.69 | - |
| Soybean oil | 2.37 | 2.91 | 1.5 |
|  | Whole grain by % from total mass, % | | |
| Wheat | - | - | 22 |
| Corn | - | - | 42.31 |
|  | Vitamin/mineral mixture by % from total mass, % | | |
| Vitamin/mineral mixture | 5.03 | 6.17 | 2.99 |
